# Prolonged morning wake transitions associated with amyloid beta burden: A cross‐sectional pilot study

**DOI:** 10.1002/alz.71123

**Published:** 2026-04-06

**Authors:** Georg von Fingerhut, Keitaro Makino, Yosuke Osuka, Kengo Ito, Takashi Kato, Shosuke Satake, Hiroyuki Shimada

**Affiliations:** ^1^ Department of Frailty Research Center for Gerontology and Social Science, National Center for Geriatrics and Gerontology Obu Aichi Japan; ^2^ Department of Preventive Gerontology Center for Gerontology and Social Science, National Center for Geriatrics and Gerontology Obu Aichi Japan; ^3^ Center for Environmental and Health Sciences Hokkaido University Sapporo Hokkaido Japan; ^4^ National Center for Geriatrics and Gerontology Obu Aichi Japan; ^5^ Department of Clinical and Experimental Neuroimaging Center for Development of Advanced Medicine for Dementia, National Center for Geriatrics and Gerontology Obu Aichi Japan

**Keywords:** amyloid beta, cognitive function, lifestyle, older adults, sleep, time to wake up

## Abstract

**INTRODUCTION:**

Sleep quality critically influences amyloid beta (Aβ) clearance, yet the specific role of morning wake transition duration remains unexplored.

**METHODS:**

This hypothesis‐driven secondary analysis examined 97 cognitively unimpaired older adults (mean age ± SD: 74.4 ± 5.8 years) who underwent 14‐day actigraphy and 18F‐florbetapir positron emission tomography (PET). Cognitive function was evaluated using the National Center for Geriatrics and Gerontology‐Functional Assessment Tool (NCGG‐FAT). Time to wake up (TWU) was calculated as the midpoint between initial wake and final rise times.

**RESULTS:**

Binomial logistic regression models adjusted for age, depression, and sleep duration showed that extended TWU (>6.23 min) associated with higher Aβ positivity (27.1% vs 10.2%, odds ratio [OR] = 1.85, 95% confidence interval [CI]: 1.11–3.07, *p* = 0.019) and impaired word memory (OR = 2.17, 95% CI: 1.11‐4.24, *p* = 0.023). Exploratory analyses of additional cognitive domains showed no significant associations.

**DISCUSSION:**

Prolonged morning wake transitions may represent a behavioral marker associated with Aβ accumulation and memory impairment. The cross‐sectional design and modest sample size warrant cautious interpretation and replication in larger longitudinal cohorts.

## Highlights

- Extended morning wake transitions (>6.23 min) are associated with a higher amyloid beta (Aβ) burden.
- Prolonged wake transition links to memory impairment independent of total sleep duration.
- Time to wake up represents a novel behavioral marker that is measurable via actigraphy.
- Wake transitions assessment could provide an accessible screening tool for Alzheimer's disease risk.

## 1 BACKGROUND

Alzheimer's disease (AD) affects more than 55 million people globally, with sleep disruption emerging as both a risk factor and early symptom.^1^ Recent evidence demonstrates bidirectional relationships between sleep and amyloid beta (Aβ) pathology: poor sleep accelerates Aβ accumulation, whereas Aβ burden disrupts sleep architecture.^2^, ^3^ Most research has focused on total sleep duration or nighttime fragmentation.

The sleep–wake transition represents a unique neurobiological state. The glymphatic system—responsible for clearing metabolic waste including Aβ—shows markedly different activity between the sleep and wake states.^4^ Recent evidence demonstrates that glymphatic clearance varies significantly across sleep–wake states. Vinje et al.^5^ showed that sleep deprivation reduces advective brain clearance by 50% in humans. More controversially, Miao et al.^6^ found that brain clearance may actually be reduced during sleep compared to wakefulness in mice. These findings raise questions about how sleep–wake transitions may relate to clearance mechanisms, although the specific role of morning transitions remains to be established.

The duration of the morning wake transition—time to wake up (TWU), calculated as the midpoint between initial wake and final rise time^7^—may serve as a behavioral marker of arousal system dysfunction. The locus coeruleus–norepinephrine (LC‐NE) system, which regulates both sleep–wake transitions and glymphatic function during sleep,^8^, ^9^ shows early involvement in AD progression. Extended morning transitions could therefore reflect cumulative LC‐NE dysfunction associated with impaired overnight clearance efficiency, although this relationship remains unexplored.

We designed this hypothesis‐driven study to test two primary hypotheses in community‐dwelling older adults: (1) extended morning wake transition duration (TWU) associates with Aβ accumulation, and (2) extended TWU associates with episodic memory impairment, the earliest cognitive manifestation of Aβ accumulation and AD pathology. We included a comprehensive cognitive battery to characterize the broader cognitive profile, hypothesizing that associations would be most evident in memory given its established sensitivity to Aβ‐related neurodegeneration in preclinical stages. 

## 2 METHODS

### 2.1 Study design, setting, and participants

This cross‐sectional study analyzed data from the National Center for Geriatrics and Gerontology Aβ imaging substudy, conducted in Obu, Takahama, and Tokai cities in Aichi Prefecture, Japan.

Participants were recruited between September 2017 and December 2019, with positron emission tomography (PET) and cognitive tests performed between October 2017 and January 2020.^10^ From an initial community‐dwelling population, we selected 97 individuals 60–90 years of age who did not meet our exclusion criteria, as: (1) neurological disorders including dementia, Parkinson's disease, multiple cerebral infarction, Huntington's disease, normal pressure hydrocephalus, brain tumor, progressive supranuclear palsy, epilepsy, subdural hematoma, multiple sclerosis, or head injury with sequelae; (2) active infection or acute cerebral infarction detected on magnetic resonance imaging (MRI); (3) contraindications to MRI due to metallic implants; (4) psychiatric conditions (major depression, bipolar disorder), substance dependence (alcohol or drugs), or metabolic disorders (vitamin B12 deficiency, syphilis, thyroid dysfunction); (5) current psychoactive medication use; (6) inability to maintain supine positioning during procedures; (7) certified care needs under the long‐term care insurance system; or (8) poor glycemic control (glycated hemoglobin [HbA1c] ≥6.5%).^11^, ^12^

The parent cohort study protocol (registration ID: UMIN000030319) was registered prospectively with the University Hospital Medical Information Network Clinical Trials Registry (available at: http://www.umin.ac.jp/ctr/index.htm).

### 2.2 Assessment

Prior to study initiation, all personnel received comprehensive training on assessment protocols. All evaluations were conducted by trained health care professionals at local community centers through structured interviews.

#### 2.2.1 Sleep assessment

Sleep parameters were measured using wrist actigraphy (Silmee W20, TDK Corporation Tokyo, Japan), recording activity in 1‐min epochs during 14 days. The wristband uses a three‐axis accelerometer to detect movement and sleep duration. The device was validated through comparison with video observation data, showing strong correlations for activity detection (*r* = 0.9869, *p* < 0.0001) and sleep duration (*r* = 0.9995, *p* < 0.0001).^13^ Although the manufacturer does not disclose the exact numerical values, the Silmee W20 wrist sensor's proprietary sleep and activity count thresholds have been described in prior validation reports.^14^

**RESEARCH IN CONTEXT**

1. **Systematic review**: Authors reviewed PubMed for studies on sleep transitions and amyloid beta (Aβ). Although extensive research exists on total sleep duration, no studies specifically examined morning wake transition duration (TWU) and its relationship to Aβ accumulation.
2. **Interpretation**: Our findings demonstrate that prolonged TWU (>6.23 min) is associated with increased Aβ burden and memory impairment, independent of total sleep duration, suggesting that wake transitions represent a novel behavioral marker.
3. **Future directions**: Longitudinal studies with polysomnography, apolipoprotein E (*APOE*) genotyping, and larger samples are needed to establish causality and clinical utility of TWU assessment.

Furthermore, participants completed detailed sleep questionnaires covering their typical sleep patterns over the previous month to complement objective measurements. These questionnaires captured retrospective reports of typical sleep patterns rather than daily sleep logs during the actigraphy period. These assessments included bedtime, sleep onset, wake time, self‐reported wake after sleep offset latency, nocturnal awakenings, and nap duration. The assessment protocol also examined sleep‐related behaviors and symptoms, including early morning awakenings, sleep quality dissatisfaction, and hypnotic medication use. For each parameter, participants indicated frequency using the options “3 or more days a week,” “1 or 2 days a week,” “less than a day a week,” and “never.” These responses were subsequently categorized into high frequency (≥3 days/week) versus lower frequency groups for analytical purposes. We paid particular attention to sleep apnea and restless leg syndrome symptoms, using standardized questions about breathing pauses during sleep and unpleasant, restless feelings in their legs categorizing responses as either “≥3 times/week” or “other.” Furthermore, participants were asked about their sleep environment satisfaction through the following question: “Are you satisfied with your sleeping environment, including light, room temperature, noise, and bedding (pillows)?” Participants selected from responses “very satisfied,” “satisfied,” “dissatisfied,” and “very dissatisfied,” which we subsequently dichotomized into satisfied versus dissatisfied categories.

#### 2.2.2 TWU calculation

The wake transition period was defined through continuous movement detection from sleep onset to final rise time. The Silmee W20 defines nocturnal waking as ≥20 min of continuous movement between sleep onset and the end of sleep,^14^ so brief arousal (e.g., 5 min at 2:00 a.m.) would not be treated as the wake time and final rise time is identified only when sustained activity consistent with rising is detected. To assess plausibility, device‐derived average wake and rise times were compared against self‐reported typical awake and out‐of‐bed times from retrospective questionnaires. Discrepancies between self‐reported typical times and device‐derived average times were minimal across the sample, supporting the plausibility of actigraphy‐derived parameters. Device‐derived values were used for all analyses. Activity count thresholds for determining wake and rise times are proprietary to the device manufacturer but were consistently applied across all participants. TWU was calculated as the midpoint between initial wake time and final rise time, following established approaches to quantifying sleep‐wake transition periods,^7^ using the formula:

$$\mathrm{TWU}={\mathrm{wake}}_{\mathrm{time}}+\left({\mathrm{rise}}_{\mathrm{time}}-{\mathrm{wake}}_{\mathrm{time}}\right)/2)$$

TWU was calculated as a duration in minutes, representing the temporal midpoint of the morning wake period. TWU captures the period between initial detected movement (device‐defined wake) and sustained activity consistent with rising. This midpoint calculation provides a stable metric for assessing the wake transition phase, similar to validated methods used in chronobiology research.^15^

#### 2.2.3 Biological and cognitive assessments

Participants underwent Aβ‐PET imaging and cognitive testing. Aβ‐PET imaging used ^18^F‐florbetapir, with PET‐computed tomographic camera (Biograph 16 True Point TV, Siemens AG, Germany) images acquired 50‐70 min post‐injection of 370 MBq ^18^F‐florbetapir.^10^ Cerebellar reference regions were used for calculating standardized uptake value ratio (SUVRs). Global neocortical SUVR was derived from anterior/posterior cingulate, precuneus, medial orbitofrontal, temporal, and parietal regions. Two radiologists, blinded to clinical and biomarker information, independently performed visual assessment of Aβ‐PET images to determine Aβ status (Aβ+/Aβ−). Discordant classifications were resolved through consensus evaluation.

Our cognitive assessment protocol incorporated two complementary approaches: the Mini‐Mental State Examination (MMSE) and the National Center for Geriatrics and Gerontology–Functional Assessment Tool (NCGG‐FAT). The NCGG‐FAT represents a significant advancement in cognitive testing, offering a tablet‐based platform that demonstrates high test–retest reliability and moderate‐to‐high criterion‐related validity.^16^ The NCGG‐FAT provides comprehensive evaluation across four key cognitive domains: memory function, assessed through word list memory tasks; attention, measured via Trail Making Test Part A (TMT‐A); executive function, evaluated using Trail Making Test Part B (TMT‐B); and processing speed, gauged through the Symbol Digit Substitution Task (SDST). For memory assessment, we calculated scores by combining performance across three immediate memory trials with delayed recall performance, allowing a maximum potential score of 20 points. The TMT‐A and TMT‐B assessments measured completion time in seconds, providing precise quantification of attention and executive function capabilities. The SDST, with its maximum score of 90 s, offered detailed insight into processing speed abilities. To ensure appropriate classification of cognitive status, we employed standardized thresholds based on a large normative sample of 19,000 community‐dwelling older adults.^17^ Cognitive impairment was defined as performance falling below 1.5 standard deviations (SD) from age‐ and education‐adjusted norms across each NCGG‐FAT domain.^17^

#### 2.2.4 Clinical characteristics

Our protocol measured the potential confounding variables affecting sleep–wake patterns and cognitive outcomes through standardized assessment of clinical and lifestyle parameters. We documented demographic factors including age, sex, educational attainment, and current family and employment status. Clinical evaluation encompassed cardiovascular conditions, hypertension, respiratory diseases, and current medication regimens. We calculated body mass index (BMI) through standard anthropometric measurements and quantified depressive symptoms using the 15‐item Geriatric Depression Scale (GDS; range 0–15).^18^ The lifestyle assessment protocol documented alcohol consumption patterns and physical activity levels. Physical activity evaluation employed standardized questioning regarding participation in both moderate‐intensity exercise and low‐intensity physical activities. Participants reporting no engagement in either activity category met established criteria for low physical activity classification.^19^

### 2.3 Statistical analyses

Continuous variables are presented as mean ± SD or median (interquartile range [IQR; 25th–75th percentile]), whereas categorical variables are presented as counts and percentages. Statistical comparison of groups employed distribution‐appropriate testing methods: unpaired *t*‐tests for normally distributed continuous variables, Mann–Whitney *U* tests for non‐parametric continuous variables, and chi‐square and Fisher's exact test analyses for categorical data accordingly. Baseline characteristics were initially explored according to TWU, followed by classifying participants into two groups according to their median split of TWU values (≤6.23 min = 0, >6.23 min = 1); the ≤6.23 min group served as the reference category.

Using the *t*‐test, chi‐square test, and adjusted standardized residuals, we determined whether the TWU duration had a significant association with Aβ. In addition, we examined the association between TWU in the two groups and the results of each cognitive tests with moderate (1.5 SD below) level of severity of cognitive impairment. For MMSE, a cutoff score of 23/24 was implemented due to better sensitivity/specificity balance.^20^

Odds ratios (ORs) and 95% confidence intervals (CIs) were estimated from the logistic models. The fully adjusted models included the following covariates: age, sex, GDS score, and sleep duration, according to previous studies.^21^ For our hypothesis‐driven analyses, statistical significance was set at *p* < 0.05 for Aβ positivity (primary hypothesis) and word memory impairment (key secondary hypothesis). No correction for multiple comparisons was applied to these two theoretically‐motivated outcomes, consistent with standard practice for hypothesis‐driven confirmatory research. Supplementary continuous analyses examined the relationships between TWU and cognitive outcomes using two approaches: (1) linear regression for continuous SUVR values adjusted for age, sex, GDS, and sleep duration; and (2) Pearson partial correlations controlling for sex between TWU (continuous, minutes) and cognitive outcomes. Spearman's correlation was selected given the non‐normal distributions of some variables. Exploratory analyses of additional cognitive domains (MMSE, TMT‐A, TMT‐B, and SDST) are reported with multiple comparison correction using the Benjamini–Hochberg false discovery rate (FDR) method.^22^ Both uncorrected and FDR‐corrected *p*‐values are reported for all exploratory analyses. Statistical analyses were performed using SPSS, an IBM Corp. program (version 29.0.1, Chicago, IL, USA).

## 3 RESULTS

### 3.1 Participant characteristics

Our study population comprised 97 community‐dwelling older adults (median age 73.0 years; IQR 69.5, 78.0), with women representing 46.4% of participants. The mean MMSE score of 26.4 ± 2.7 indicated generally preserved cognitive function at baseline. When comparing the TWU groups, we found comparable baseline characteristics across most measures, with significant difference emerging only in Aβ‐deposition patterns (Table 1).

**TABLE 1:** Baseline characteristics of participants based on time to wake up.

	All participants	TWU ≤6.23 min	TWU >6.23 min	*p* Value
	*n* = 97	*n* = 49	*n* = 48	
Age	73.0 [69.5, 78.0]	75.0 [70.0, 80.0]	71.0 [69.0, 77.8]	0.142^†^
Women, *n* (%)	45 (46.4)	22 (22.7)	23 (23.7)	0.840
BMI, kg/m^2^	23.5 ± 3.4	23.1 ± 3.5	24.1 ± 3.1	0.185
Education, years	12.0 [9.0, 12.0]	12.0 [9.0, 12.0]	12.0 [9.3, 13.5]	0.326^†^
MMSE, points	27.0 [25.0, 29.0]	26.0 [24.0, 29.0]	26.0 [25.0, 29.0]	0.705^†^
GDS, points	2.0 [1.0, 4.0]	2.0 [1.0, 4.0]	2.0 [1.0, 4.5]	0.738^†^
Aβ deposition, *n* (%)	18 (18.6)	5 (5.2)	13 (13.4)	0.039
Cardiovascular diseases, *n* (%)	8 (8.2)	6 (6.2)	2 (2.1)	0.268
Hypertension, *n* (%)	46 (47.4)	26 (26.8)	20 (20.6)	0.312
Respiratory diseases, *n* (%)	12 (12.5)	7 (7.3)	5 (5.2)	0.759
Medications, *n*	2.0 [1.0, 3.0]	2.0 [1.0, 3.0]	2.0 [1.0, 3.0]	0.093^†^
Working, *n* (%)	30 (30.9)	15.0 (15.5)	15 (15.5)	1.000
Alcohol drinking, *n* (%)	43 (44.3)	23.0 (23.7)	20 (20.6)	0.684
Low physical activity, *n* (%)	16 (16.5)	6.0 (6.2)	10 (10.3)	0.286

TWU, time to wake up; BMI, body mass index; FDR, false discovery rate; GDS, Geriatric Depression Scale; MMSE, Mini‐Mental State Examination. Values are expressed as mean ± SD, median [IQR; 25th–75th percentile], or n (%), as appropriate. *t*‐Test,

^†^ Mann–Whitney *U* test. *p*‐values shown are uncorrected. FDR‐corrected values are reported in text and supplementary materials.

### 3.2 Sleep parameters and Aβ accumulation

The proportion of participants with Aβ+ was significantly higher in the extended TWU group (13 of 48, 27.1%) compared to the shorter TWU group (5 of 49, 10.2%, *p* = 0.039). Beyond binary classification, participants with extended TWU showed higher global SUVR values (1.18 ± 0.14 vs 1.09 ± 0.11, *p* = 0.031, Cohen's *d* = 0.71). Effect sizes were moderate (Cohen's *d* = 0.52 for Aβ,*d* = 0.48 for memory).

Sleep parameter analysis revealed contrasts between groups. Participants with extended TWU showed notably shorter sleep onset latency (10.6 ± 7.8 vs 17.1 ± 19.4 min, *p* < 0.001). Sleep duration did not differ between groups (shorter TWU: 443.3 ± 66.3 min vs extended TWU: 429.2 ± 60.0 min, *p* = 0.520) (eTable 1). The histogram of TWU is shown in eFigure 1.

### 3.3 Primary and secondary outcomes

Logistic regression analyses, adjusted for age, GDS score, and sleep duration, revealed that extended TWU was significantly associated with our primary outcome of Aβ positivity (OR = 1.85, 95% CI: 1.11–3.07, *p* = 0.019), and our key secondary outcome of word memory impairment (OR = 2.17, 95% CI: 1.11–4.24, *p* = 0.023). Sex was not significantly associated with any outcome (all *p*’s > 0.16). In supplementary continuous analyses, linear regression confirmed that TWU significantly predicted global SUVR (β = 0.034, 95% CI: 0.005–0.064, *p* = 0.024) after adjustment for age, sex, GDS, and sleep duration (eTable 2a). Partial correlation controlling for sex between TWU and SUVR was *r* = 0.239, *p* = 0.019 (eTable 2b).

**FIGURE 1:**
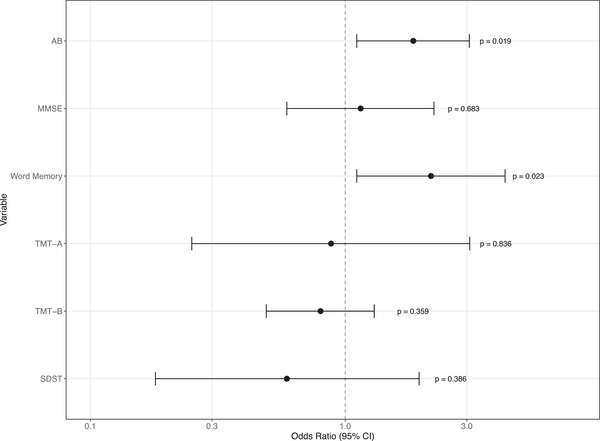
Results of logistic regression analysis of associations of time to wake up, amyloid beta, and cognitive scores.

Exploratory analyses of additional cognitive domains showed that no significant associations were observed between extended TWU and other cognitive measures: MMSE (OR = 1.15, 95% CI: 0.59–2.23, *p* = 0.683, TMT‐A (OR = 0.88, 95% CI: 0.25–3.08, *p* = 0.836), TMT‐B (OR = 0.80, 95% CI: 0.49–1.30, *p* = 0.359), or SDST (OR = 0.59, 95% CI: 0.18–1.95, *p* = 0.386) (Figure 1). Supplementary partial correlations controlling for sex demonstrated that TWU showed a moderate positive correlation with word memory impairment (*r* = 0.320, *p* = 0.001; eTable 2b). Exploratory partial correlations with other cognitive outcomes (TMT‐A, TMT‐B, SDST) remained nonsignificant after FDR correction (all FDR *p* = 0.444; eTable 2c), providing convergent evidence across different analytical approaches (eTable 2).

## 4 DISCUSSION

This hypothesis‐driven secondary analysis examined associations between actigraphy‐measured morning wake transition duration (TWU) and Aβ burden in 97 cognitively unimpaired older adults. Extended TWU (>6.23 min, median split) was significantly associated with Aβ positivity (OR = 1.85, *p* = 0.019) and word memory impairment (OR = 2.17, *p* = 0.023) after adjusting for age, sex, depression, and sleep duration. Continuous analyses confirmed a dose–response relationship between TWU and global amyloid burden (SUVR: β = 0.034, *p* = 0.024). The TWU–memory association was robust across analytical approaches: partial correlation analysis controlling for sex confirmed this relationship (*r* = 0.320, *p* = 0.001). Exploratory analyses of additional cognitive domains showed no significant associations. These findings suggest that TWU may serve as an accessible behavioral marker associated with early Aβ accumulation and memory dysfunction.

### 4.1 Main findings and mechanisms

Our findings align with and extend recent large‐scale evidence on sleep's role in Aβ accumulation. In a study of 4425 cognitively unimpaired older adults, Insel et al.^1^ demonstrated that each additional hour of nighttime sleep was associated with reduced Aβ deposition, particularly in the medial orbitofrontal (0.009 reduction) and anterior cingulate regions (0.011 reduction). Recent work by Hauglund et al.^23^ identified norepinephrine‐mediated slow vasomotion as the primary driver of glymphatic clearance during non–rapid eye movement (NREM) sleep, with oscillations at ≈0.02 Hz creating rhythmic pumping of cerebrospinal fluid. Particularly, disruption of these oscillations—which could occur during extended wake transitions—reduces clearance by >30%.^23^ It is notable that their study showed these associations were present even before significant Aβ accumulation, supporting our hypothesis that sleep–wake transitions may represent an early intervention target. Although Insel's study^1^ focused on total sleep duration, our findings specifically highlight the role of morning wake transitions, suggesting a potential circadian component to Aβ clearance mechanisms. These complementary results underscore the importance of both sleep quantity and timing in modulating Aβ pathology. The observed association between TWU and Aβ positivity (OR = 1.84) is modest compared to genetic risk factors like apolipoprotein E (*APOE*) ε4 (typically OR >3), but comparable to other modifiable lifestyle factors associated with AD risk, suggesting potential clinical relevance if confirmed longitudinally.

Several mechanisms may explain the association between extended TWU and Aβ accumulation. Extended TWU may serve as a marker of cumulative arousal system dysfunction, although causal relationships cannot be established from cross‐sectional data. The locus coeruleus–norepinephrine system, crucial for sleep–wake regulation and showing early involvement in AD progression, may contribute to extended wake transitions.^8^, ^9^ In addition, age‐related changes in neurotransmitter systems including γ‐aminobutyric acid (GABA), which correlate with cognitive performance in older adults,^24^ may play a role, although specific relationships with TWU require further investigation.

### 4.2 Methodological considerations and temporal dynamics

Our TWU midpoint calculation approach differs from wakefulness after sleep‐offset latency measurement but provides a complementary metric capturing the temporal center of the entire wake transition window. By quantifying the temporal center of the entire wake transition window rather than absolute wake or rise times, this approach reduces sensitivity to day‐to‐day variability in sleep timing caused by social factors (e.g., weekend vs weekday schedules), providing a more stable individual‐level metric across our 14‐day measurement period. This approach parallels chronobiological metrics such as mid‐sleep, which have been widely validated as markers of circadian alignment and social jetlag.^15^, ^25^ By doing so, we achieve more reliable measurements of the wake transition period than in our previous studies on morning wake transition.^26^ Understanding these temporal windows could prove crucial for developing time‐sensitive interventions.

### 4.3 Study limitations and interpretation

Our study has several important limitations requiring cautious interpretation.

First, the cross‐sectional design precludes causal inference. Reverse causation—where subclinical Aβ pathology disrupts arousal systems leading to extended TWU—remains equally plausible. Only longitudinal studies tracking changes in both TWU and Aβ burden over time can determine whether TWU changes precede, follow, or develop concurrently with Aβ accumulation.

Second, our sample size (*n* = 97) provides 60% power to detect the observed effect sizes, falling below conventional 80% thresholds. The modest ORs (Aβ: OR = 1.85; memory: OR = 2.17) and wide CIs indicate substantial uncertainty in effect estimates. The lack of significant associations between TWU and exploratory cognitive outcomes (MMSE, TMT‐A, TMT‐B, and SDST) may reflect true absence of associations for these domains, insufficient statistical power, or measurement limitations. These exploratory findings require replication in adequately powered studies before interpretation.

Third, critical unmeasured confounders limit interpretation. Without *APOE* ε4 genotyping, we cannot determine whether TWU–Aβ associations differ by genetic risk, as demonstrated in recent Australian Imaging, Biomarkers and Lifestyle (AIBL) cohort findings.^27^ In addition, we lacked polysomnographic data to characterize sleep architecture or confirm actigraphy‐derived parameters. The manufacturer's validation of the Silmee W20 against video observation rather than polysomnography may limit sleep–wake detection accuracy. Of note, actigraphy detects movement thresholds rather than EEG‐defined sleep–wake states. TWU therefore captures time between detected movement onset and rising behavior, which may include both biological arousal transition time and subsequent time spent in bed before rising. We cannot distinguish whether extended TWU reflects prolonged arousal transitions, volitional rest after waking, or both. Future studies should incorporate polysomnography to characterize neurophysiological arousal states, and morning behavior diaries to capture specific activities (e.g., reading, phone use, or quiet rest) during the wake‐to‐rise period. In addition, sleep questionnaires captured typical patterns retrospectively rather than through daily logs, thereby limiting day‐by‐day validation of actigraphy‐derived parameters.

### 4.4 Clinical translation and future directions

If validated longitudinally, TWU assessment could provide an accessible, non‐invasive screening tool. Actigraphy costs <$200 compared to >$5000 for Aβ‐PET. Although plasma Aβ biomarkers represent the emerging clinical standard for AD detection, actigraphy‐based TWU assessment may provide a complementary functional marker that captures behavioral manifestations of early pathology at a substantially lower cost. Potential interventions might include bright light therapy to enhance morning arousal, scheduled morning activities to reduce wake transition time, and pharmacological optimization of arousal systems. However, implementation requires establishing normal TWU ranges across diverse populations and determining modification feasibility in at‐risk individuals, and conducting methodological studies incorporating polysomnography to characterize neurophysiological arousal states and morning behavior diaries to distinguish biological transitions from volitional in‐bed activities.

These research directions may inform future intervention studies targeting this critical period, potentially modifying AD risk through optimized wake transitions. Translation into clinical practice requires careful consideration of individual circumstances, with morning wake patterns potentially serving as an accessible behavioral marker for monitoring brain health.

## 5 CONCLUSION

Extended morning TWU was associated with increased Aβ burden and memory impairment in older adults. Although these findings suggest TWU may be relevant to AD pathophysiology, the cross‐sectional design limit causal interpretations. Longitudinal studies incorporating chronotype, sleep architecture, and genetic factors are needed to establish whether optimizing morning wake patterns could modify AD risk.

## CONFLICT OF INTEREST STATEMENT

All authors declare no competing interests. Detailed conflict of interest disclosures are available in the supplementary documentation. Author disclosures are available in the Supporting Information.

## CONSENT STATEMENT

Participant data were only utilized after obtaining their explicit consent. The Ethics Committee of the National Center for Geriatrics and Gerontology approved the study protocol (approval number 1440‐5) after obtaining informed consent from all participants prior to their inclusion in the research.

## Supporting information



Supporting Information

Supporting Information

Supporting Information

Supporting Information

## Data Availability

Data from this investigation are not publicly accessible due to institutional privacy regulations protecting participant confidentiality. No study pre‐registration was conducted.
