# Supplementary material for: Prolonged morning wake transitions associated with amyloid beta burden: A cross‐sectional pilot study
Source: Alzheimers Dement. 2026 Apr 6;22(4):e71123. doi: 10.1002/alz.71123 (PMC13053936; doi:10.1002/alz.71123)
Supplement: Supplementary file 1 — Supporting Information [file ALZ-22-e71123-s002.doc]

**eFigures**

eFigure 1. Distribution of time to wake up values in study participants.

Histogram showing the distribution of TWU (time to wake up) values among 97 study participants. TWU represents the temporal midpoint of the morning wake period in minutes.
