# Supplementary material for: Prolonged morning wake transitions associated with amyloid beta burden: A cross‐sectional pilot study
Source: Alzheimers Dement. 2026 Apr 6;22(4):e71123. doi: 10.1002/alz.71123 (PMC13053936; doi:10.1002/alz.71123)
Supplement: Supplementary file 2 — Supporting Information [file ALZ-22-e71123-s003.doc]

**Supplementary**

eTable 1. Sleep characteristics of the study participants (n = 97)

|  | **All participants** | **TWU ≤6.23 min** | **TWU >6.23 min** | ***p*-value** |
| --- | --- | --- | --- | --- |
|  | n = 97 | n = 49 | n = 48 |
| **Sleep onset latency, min** | 10.0 [5.0, 20.0] | 10.0 [5.0, 30.0] | 10.0 [5.0, 10.0] | <.001 † |
| **Sleep duration, min** | 437.6 ± 63.6 | 443.3 ± 66.3 | 429.2 ± 60.0 | 0.661 |
| **Self-reported wake after sleep offset latency, min** | 5.0 [1.0, 30.0] | 5.0 [0.0, 20.0] | 9.0 [5.0, 30.0] | 0.506 † |
| **Nocturnal awakenings, times per night** | 1.0 [1.0, 2.0] | 1.0 [1.0, 2.0] | 2.0 [1.0, 2.0] | 0.476 † |
| **Nap duration, min** | 30.0 [15.0, 45.0] | 30.0 [20.0, 60.0] | 30.0 [15.0, 41.3] | 0.352 † |
| **Early morning awakenings ≥3 time per week, n (%)** | 4 (4.1%) | 0 (0.0%) | 4 (4.1%) | 0.056 ‡ |
| **Self-reported symptoms of apnea ≥3 time per week, n (%)** | 3 (3.1%) | 2 (2.1%) | 1 (1.0%) | 1.000 ‡ |
| **Self-reported symptoms of RLS ≥3 time per week, n (%)** | 3 (3.1%) | 1 (1.0%) | 2 (2.1%) | 0.617 ‡ |
| **Sleeping pills use ≥3 times per week, n (%)** | 2 (2.1%) | 2 (2.1%) | 0 (0.0%) | 0.495 ‡ |
| **Sleep quality dissatisfaction, n (%)** | 4 (4.1%) | 0 (0.0%) | 4 (4.1%) | 0.056 ‡ |
| **Sleep environment dissatisfaction, n (%)** | 1 (1.0%) | 0 (0.0%) | 1 (1.0%) | 0.495 ‡ |

Data are expressed as n (%) for category variables and mean ± standard deviation for continuous variables. t-test; †: Mann–Whitney U test, ‡: Fisher's exact test. TWU, time to wake up; RLS, restless leg syndrome.
