# Supplementary material for: Prolonged morning wake transitions associated with amyloid beta burden: A cross‐sectional pilot study
Source: Alzheimers Dement. 2026 Apr 6;22(4):e71123. doi: 10.1002/alz.71123 (PMC13053936; doi:10.1002/alz.71123)
Supplement: Supplementary file 3 — Supporting Information [file ALZ-22-e71123-s001.doc]

**Supplementary**

eTable 2a. Linear Regression Analysis of TWU with SUVR (n = 97)

|  | β/ρ | 95% CI | *p*-value |
| --- | --- | --- | --- |
| TWU (continuous) | 0.034 | 0.005–0.064 | 0.024 |
| Age | 0.005 | -0.001–0.011 | 0.082 |
| Sex | 0.019 | -0.056–0.094 | 0.620 |
| GDS | -0.008 | -0.020–0.004 | 0.203 |
| Sleep duration | 0.000 | 0.000–0.001 | 0.130 |

Model R²=0.126, F(5,91)=2.62, p=0.029 TWU, time to wake up; SUVR, standardized uptake value ratio; GDS, geriatric depression scale.

eTable 2b. Partial Correlations of TWU with Primary Outcomes (controlling for sex)

|  | r | p-value |
| --- | --- | --- |
| TWU vs SUVR | 0.239 | 0.019 |
| TWU vs Word Memory Impairment | 0.320 | 0.001 |

TWU, time to wake up; SUVR, standardized uptake value ratio.

eTable 2c. Partial Correlations of TWU with Exploratory Cognitive Outcomes (controlling for sex)

|  | r | p-value | FDR-corrected p-value |
| --- | --- | --- | --- |
| TWU vs TMT-A Impairment | -0.079 | 0.444 | 0.444 |
| TWU vs TMT-B Impairment | -0.106 | 0.303 | 0.444 |
| TWU vs SDST Impairment | -0.122 | 0.235 | 0.444 |

TWU, time to wake up; TMT, Trail Making Test; SDST, Symbol Digit Substitution Task.

Legend: Supplementary continuous analyses examining relationships between TWU and outcomes. (a) Linear regression adjusted for age, sex, GDS, and sleep duration. (b) Pearson partial correlations controlling for sex for primary outcomes (Aβ burden, word memory). (c) Pearson partial correlations controlling for sex for exploratory cognitive outcomes with Benjamini-Hochberg FDR correction. Abbreviations: TWU, time to wake up; SUVR, standardized uptake value ratio; CI, confidence interval; FDR, false discovery rate; TMT-A/B, Trail Making Test parts A/B; SDST, Symbol Digit Substitution Task.
